# The impact of aquaculture on the genetics and distribution of the onuphid annelid *Diopatra biscayensis*

**DOI:** 10.1002/ece3.7447

**Published:** 2021-05-02

**Authors:** Matthew P. Galaska, David S. Wethey, Andrés Arias, Stanislas F. Dubois, Kenneth M. Halanych, Sarah A. Woodin

**Affiliations:** ^1^ Cooperative Institute for Climate, Ocean, & Ecosystem Studies NOAA Pacific Marine Environmental Lab University of Washington Seattle Washington USA; ^2^ Department of Biological Sciences Auburn University Auburn Alabama USA; ^3^ Department of Biological Sciences University of South Carolina Columbia South Carolina USA; ^4^ Departamento de Biología de Organismos y Sistemas (Zoología) Universidad de Oviedo Oviedo Spain; ^5^ IFREMER – DYNECO LEBCO Plouzané France

**Keywords:** Bay of Biscay, Convex Hull, Onuphidae, phylogeography, population genetics, RADseq

## Abstract

**Aim:**

Evolutionary history of natural populations can be confounded by human intervention such as the case of decorator worm species *Diopatra* (Onuphidae), which have a history of being transported through anthropogenic activities. Because they build tubes and act as ecosystem engineers, they can have a large impact on the overall ecosystem in which they occur. One conspicuous member, *Diopatra biscayensis*, which was only described in 2012, has a fragmented distribution that includes the Bay of Biscay and the Normanno‐Breton Gulf in the English Channel. This study explores the origin of these worms in the Normanno‐Breton region, which has been debated to either be the result of a historic range contraction from a relic continuous population or a more recent introduction.

**Location:**

Northeastern Atlantic, the Bay of Biscay, and the Normanno‐Breton Gulf.

**Methods:**

We utilized a RAD‐tag‐based SNP approach to create a reduced genomic data set to recover fine‐scale population structure and infer which hypothesis best describes the *D. biscayensis* biogeographic distribution. The reduced genomic data set was used to calculate standard genetic diversities and genetic differentiation statistics, and utilized various clustering analyses, including PCAs, DAPC, and admixture.

**Results:**

Clustering analyses were consistent with *D. biscayensis* as a single population spanning the Bay of Biscay to the Normanno‐Breton Gulf in the English Channel, although unexpected genetic substructure was recovered from Arcachon Bay, in the middle of its geographic range. Consistent with a hypothesized introduction, the isolated Sainte‐Anne locality in the Normanno‐Breton Gulf was recovered to be a subset of the diversity found in the rest of the Bay of Biscay.

**Main conclusions:**

These results are congruent with previous simulations that did not support connectivity from the Bay of Biscay to the Normanno‐Breton Gulf by natural dispersal. These genomic findings, with support from previous climatic studies, further support the hypothesis that *D. biscayensis* phylogeographic connectivity is the result of introductions, likely through the regions’ rich shellfish aquaculture, and not of a historically held range contraction.

## Graphical abstract

This study explores the origin of the decorator worm species *Diopatra biscayensis* in the Normanno‐Breton Gulf, a region with a rich aquaculture history. A RAD‐tag based SNP approach was used to assess the fine scale population structure and infer whether *D. biscayensis*’ distribution was likely the result of a historic range contraction from a relic continuous population or a more recent introduction.

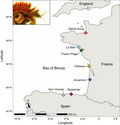

## 1 INTRODUCTION

Anthropogenic activities can make assessing historic organismal distributions challenging. Within short time intervals, humans can transport individuals across broad geographic regions, cause extinction of populations, or facilitate range shifts (Strauss et al., 2006) impacting the ability to assess population‐level dynamics. Distinguishing between changes over time due to natural causes or due to human‐mediated activities is important to the evaluation of anthropogenic environmental impacts and to aid management and conservation practices. One such example where human activities appear to have obscured biogeographic history includes decorator worm species belonging to *Diopatra (*Audouin & Milne Edwards, 1833 *),* Onuphidae. Large species of *Diopatra* have present‐day phylogeographic patterns that are particularly difficult to interpret as they have been moved for sale as fishing bait, in association with shellfish aquaculture, and have undergone natural shifts in ranges partly in response to climate change (Arias et al., 2016; Berke et al., 2010; Saito et al., 2014; van der Have et al., 2015; Wethey et al., 2016; Woodin et al., 2014).

**FIGURE 1:**
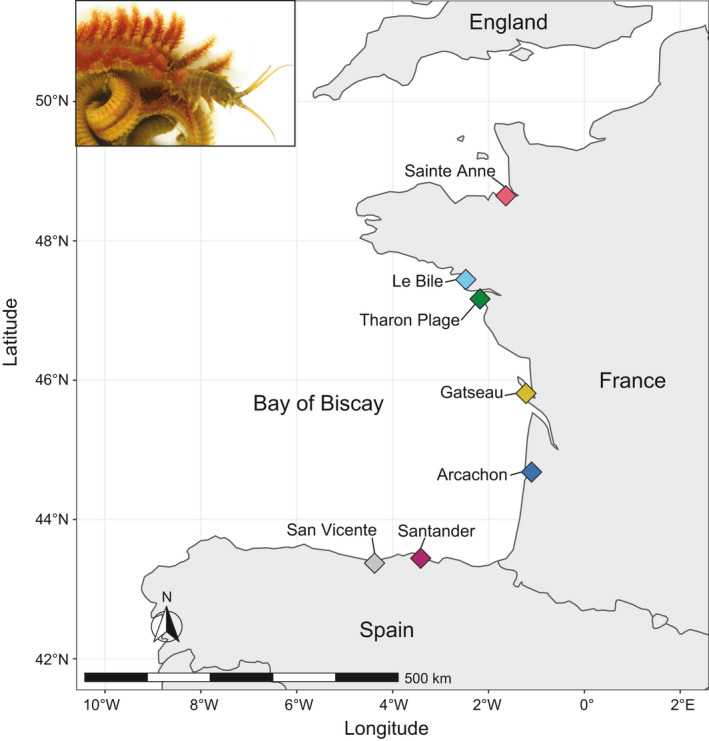
Map and image of *Diopatra biscayensis* illustrating the seven sampling localities where individuals were collected

Two conspicuous species of *Diopatra* occur along the western coastlines of France and Spain in intertidal to subtidal habitats where they build tubes decorated with debris such as shell and algal fragments. Whereas *D. neapolitana* occurs within Spanish waters and along the southern French coast, *D. biscayensis* extends from San Vicente de la Barquera, Spain (43.3833° N, 04.3833° W), to the Normanno‐Breton Gulf, near Champeaux, France (48.7327° N, 1.5521° W), on the English Channel (Arias & Paxton, 2015; Wethey et al., 2016; Woodin et al., 2014; Figure 1). The more or less contiguous distribution of *D. biscayensis* has a northern limit at La Trinité‐sur‐Mer, France, within the Bay of Biscay (47.5830°N, 3.0242°W), but approximately 450 km of coastline further into the English Channel and around the tip of Brittany, there are a number of localities hosting *D. biscayensis* within the Normanno‐Breton Gulf. What is not well understood is whether the disjunct field sites inhabited by *D. biscayensis* are the result of natural or anthropogenic causes.

The oldest historical records of *D. biscayensis* are from collections from Spanish sites in the early 1900s. These were misidentified as *D. neapolitana* at the time of collection (Arias & Paxton, 2015). Up until at least the 1930s, and probably into the 1960s, neither species was known north of Arcachon, France (44.66°N, 1.1417°W) (Berke et al., 2010; Fauchald et al., 2012; Faure, 1969). *Diopatra biscayensis* produces short‐lived gelatinous egg masses attached to its tube, which disintegrate after ~ 2 days, releasing lecithotrophic larvae that rapidly settle (Arias & Paxton, 2015). Based on larval transport simulations, the larvae are unlikely to disperse more than 50 km and probably much less (Woodin et al., 2014). However, most of the disjunct localities within the Normanno‐Breton Gulf in the English Channel (2.4°W–1.6°W, 48.6°N–48.7°N) are more than 450 km away from other known localities and are associated with extensive imports of aquaculture material (Goulletquer & Le Moine, 2002; Woodin et al., 2014). Further, the 450 km of coastline between disjunct localities, including the Brittany Peninsula, was shown in previous simulations to not be hospitable to *Diopatra* until likely the late 21st century due to cold water temperatures (Wethey et al., 2016). Important source localities for aquaculture are in the region between the Loire and Gironde rivers on the central French Biscay coast (Héral, 1989; Muehlbauer et al., 2014), areas with very large populations of *D. biscayensis* (Woodin et al., 2014).

Whereas Woodin et al. (2014) hypothesized human‐assisted transport seemed likely given the lack of evidence of stepping stone dispersal and limited larval transport probability, Arias and Paxton (2015) hypothesized that the original range of *D. biscayensis* was more extensive with a more contiguous distribution from the Mediterranean to the Normanno‐Breton Gulf. Using historical climate simulations combined with metapopulation models, Wethey et al. (2016) demonstrated that historical simulations did not support the hypothesis that *D. biscayensis* had a native range that had historically been continuous from the Mediterranean and southern Iberia to the Normanno‐Breton Gulf. The models of Wethey et al. (2016) indicated that the only suitable habitat during the last glacial maximum was along the south coast of Iberia and southern Mediterranean that recolonization of the Bay of Biscay during the last millennium (850–2,000) might have been blocked by cold regions in northwest Iberia and would not have been possible beyond the current range limit in the Bay of Biscay. Wethey et al. (2016) further argued that the climatic simulations supported the hypothesis that *D. biscayensis* was introduced into the Bay of Biscay, and the northern disjunct populations are secondary introductions from the Bay of Biscay possibly associated with aquaculture (Wethey et al., 2016, see Goulletquer & Le Moine, 2002). Currently available genetic and morphological evidence (Arias et al., 2016; Berke et al., 2010; Pires et al., 2010) is consistent with the human‐assisted transport hypothesis of Woodin et al. (2014) and Wethey et al. (2016) as is the common finding of juvenile *Diopatra* that have settled on clam boxes, ropes for seed collection, and living oyster and mussel shells (unpub.data, A. Arias and S.A. Woodin). However, available genetic data are based solely on mitochondrial, single‐locus markers, which have limited resolution over the timescale of interest. Thus, to distinguish between the human‐mediated transport hypothesis and the range reduction hypothesis (Arias & Paxton, 2015), we employed a reduced representation genome approach, specifically the 2b‐RAD method (Wang et al., 2012), to assess evolutionary patterns of population genetic variation. Notably, the two competing hypotheses differ in their predictions of isolation by distance and genetic similarity when comparing northern localities to those further south. If the disjunct northern worms represent a relic of a formerly continuous population, one would expect the genetic signature to reflect that isolation given the multiple generations necessary to traverse over 450 km of coastline given a very short larval period. Alternatively, if the disjunct northern locality is the result of a recent introduction via human‐assisted transport, then the expectation is of a genetic signature congruent with, and a subset of, the haplotype diversity recovered from the contiguous population of the Bay of Biscay.

## 2 METHODS

### 2.1 Sample collection

Only two large species of *Diopatra* are known from the Atlantic coast of Europe. *Diopatra biscayensis* is an onuphid polychaete that builds a very conspicuous tube that extends above the sediment surface (i.e., “tube‐cap”), and it is heavily decorated with debris such as shell fragments, and the aperture typically points downward (Wethey et al., 2016). Its congener *D. neapolitana,* with a similar but more southerly range, has a tube with a more limited extension above the sediment surface, minimal decoration, much thicker interior tube lining, and upward‐pointing tube aperture (Arias et al., 2016).

Approximately 20–30 individuals of *D. biscayensis* were taken by shovel from each collection locality by the authors, except those from Santander, which were obtained by AA from bait sellers who had collected them that morning (Figure 1, Table 1). SAW and DSW collected from Arcachon to Tharon Plage on the French coast, SFD collected from Sainte‐Anne in the Bay of Mont‐Saint‐Michel within the Normanno‐Breton Gulf, and AA collected on the Spanish coast from San Vicente de la Barquera. After all individuals were obtained from a site, worms were removed from their tubes, cleaned of particulate matter in seawater, and placed into individually labeled tubes containing 100% molecular grade ethanol. After 24–36 hr, antennae (or branchiae if the head was missing) were clipped and placed into a separately labeled vial with ethanol and the worm was returned to its labeled vial with new alcohol. For 21 specimens from Santander, heads were obtained; median or posterior portions were available for the other 7. In 24 individuals from San Vicente de la Barquera estuary, heads were available; the remaining 15 had only median or posterior portions. After collections, AA and SAW confirmed worm identifications by examination of diagnostic features including presence/absence of double postchaetal lobes and number of teeth on pectinate chaetae; *D. biscayensis* possess double postchaetal lobes on anterior chaetigers and pectinate chaetae with 9–32 teeth, while *D. neapolitana* lacks double postchaetal lobes and has only 5–10 teeth on pectinate chaetae (Arias & Paxton, 2015; Arias et al., 2016; Fauchald et al., 2012). Samples were sent to Auburn University for molecular characterization where KMH and Viktoria Bogantes again verified tooth count.

**TABLE 1:** Collection site details including coordinates, collection date, and the total number of worms available for analysis

Location name	Coordinates	Collection date	Number of worms	*H*_o_	*H*_e_	*F*_is_	*F*_is_ (ll)	*F*_is_ (ul)
San Vicente de la Barquera, Bay of Biscay	43.383°N, 04.383°W	March 9, 2012	39	0.222	0.261	0.162	0.151	0.172
Santander, Bay of Biscay	43.450°N, 03.417°W	September 29, 2015	24	0.218	0.261	0.187	0.176	0.199
Arcachon, Bay of Biscay	44.658°N, 1.143°W	June 3, 2016	31	0.219	0.254	0.154	0.144	0.165
Gatseau, Bay of Biscay	45.812N, 1.220°W	June 4, 2016	29	0.226	0.263	0.157	0.147	0.168
Tharon Plage, Bay of Biscay	47.165°N, 2.168°W	June 7, 2016	31	0.220	0.257	0.160	0.150	0.172
Le Bile, Bay of Biscay	47.445°N, 2.475°W	June 8, 2016	28	0.224	0.261	0.160	0.149	0.171
Sainte‐Anne, Bay of Mont‐Saint‐Michel	48.647°N, 1.647°W	August 21, 2016	27	0.214	0.260	0.196	0.183	0.208

Additionally, genetic diversity indices observed heterozygosity (*H*_o_), expected heterozygosity (*H*_e_), inbreeding coefficient (*F*_is_), and the lower limit (ll) and upper limit (ul) confidence intervals calculated from 1,000 bootstrap replicates are also included.

### 2.2 Genomic data

DNA was extracted from individuals of *D. biscayensis* using an antenna or branchia using the Qiagen DNeasy Blood & Tissue Kit following manufacturer's protocols. The quality of the DNA was subsequently checked on a 1% TAE gel and a Qubit 2.0 Fluorometer. The 2b‐RAD protocol of Wang et al. (2012), which employs the restriction enzyme *Alf*I, was used to generate a reduced representation library (using a 1/16th adaptor ligation reduction scheme). Sample libraries were dual‐barcoded and sent to Hudson Alpha Institute for Biotechnology (Huntsville, Alabama) for sequencing on an Illumina HiSeq using 50 bp single‐end chemistry.

Raw sequence reads were demultiplexed and quality‐filtered to remove any reads with less than 90% of nucleotides having a Phred score above 20. Additionally, reads were filtered for the presence of the *Alf*I recognition site (scripts from https://github.com/Eli‐Meyer/2brad_utilities). Retained filtered sequences were then assembled using the Stacks v 2.1 (Catchen et al., 2011) *denovo_map.pl* pipeline with most parameters left in the default setting (except for distances allowed between stacks, *M* = 3, and distance allowed between catalog loci, *n* = 3, based on recommendations by Paris et al. (2017)). Retained loci were then processed in the *populations* program of the Stacks package with a minimum minor allele frequency of 0.05, a maximum observed heterozygosity of 0.5, and retaining only one SNP per RAD locus with the write_single_snp command. To reduce the amount of missing data, a standard filtering approach (Benestan et al., 2015; Galaska et al., 2017b; Paris et al., 2017) was applied. A given locus had to be present in at least 70% of individuals at a given sampling locality and in five of the seven localities to be retained. This filtering scheme was selected to remove any loci that did not have adequate sequencing coverage across the sample set and to mitigate the effects that allele dropout can have on estimating variation within and between populations (Gautier et al., 2013). The resulting SNP matrix was then exported in “Structure” format for downstream analyses based on the allelic variation. Analyses that required different data formats were converted through the program *PGDSpider* v2.0.8.1 (Lischer & Excoffier, 2012).

### 2.3 Genetic diversity, population structure, and gene flow

The resulting SNP matrix was imported into the R v3.4.2 statistical environment (R Core Team, 2017) for further filtering and population genetic inference. *BayeScan* v.2.01 (Foll & Gaggiotti, 2008) was used with a 50,000 interval burn‐in, 100,000 intervals, and priors of 1,000 and 10,000 to identify any loci potentially under selection. Identification of putative loci under possible selection was attempted using a *blastn* analysis against the NCBI nucleotide database.

Tests of standard genetic distances and general nucleotide diversity indices were also calculated in the R statistical environment v 3.4.2 (R Core Team, 2017) with the package *hierfstat* v0.04–22 (Goudet, 2005). The statistic “Dch” or the Cavalli‐Sforza and Edwards Chord distance (Cavalli‐Sforza & Edwards, 1967) was selected for its increased accuracy in estimating relationships among samples, in comparison with other measures of genetic distance (Takezaki & Nei, 1996). A pairwise *F*_st_ estimate using Weir and Cockerham (1984) statistic was also calculated and then bootstrapped for 1,000 replicates to provide upper and lower limit confidence intervals. To calculate signals of isolation by distance (IBD), the Mantel tests were performed in the *ade4* v1.7–13 (Dray & Dufour, 2007) software package using the Cavalli‐Sforza and Edwards Chord genetic distance values calculated between localities. Analyses of molecular variance (AMOVAs) were calculated in the *poppr* v2.8.1 software package (Kamvar et al., 2015). Significance testing was done by the randtest function in *ade4* v1.7–13 in R (Dray & Dufour, 2007). Clustering‐based analyses, such as principal component analyses, along with discriminant analysis of principal components (DAPC), were calculated within the *adegenet* v2.1.1 software package (Jombart, 2008; Jombart & Ahmed, 2011; Jombart et al., 2010). The optimal number of principal components that *adegenet* retains was selected by using the *adegenet's* cross‐validation xvalDapc command. *Adegenet's* DAPC then uses a Bayesian information criterion to assess the “true” value of *K* or number of populations present in the data. Convex hull analyses were performed on PCAs using the R software package *grDevices* v3.4.2 (R Core Team, 2017) to estimate total variation of a given locality as represented by PCA. The program *Admixture* v1.3.0 (Alexander et al., 2009) was also used to estimate the true value of *K* and to estimate admixture rates from ancestral populations. To assess gene flow patterns across our study area, the program *divMigrate* of the *diversity* R (Keenan et al., 2013) package was used to estimate relative migration rates and directionality between sampling localities using Nei's *G*st method (Nei, 1973; Nei & Chesser, 1983).

All analyses were done after filtering of loci and individuals. The number of individuals in Table 1 reflects the 209 available for analysis of the 229 individuals collected. Several specimens were lost due to degraded DNA before the RADseq prep, a few due to low sequencing and several due to low coverage of loci postfiltering. All such individuals were removed from the entire analysis pipeline so that individuals with missing data did not impact downstream filtering or further analyses.

## 3 RESULTS

### 3.1 Genomic data set generation and filtering

The data set consisted of 209 individuals coded for 4,078 polymorphic SNP loci with an average sequencing depth of 51.4x, after filtering all RAD loci and samples for quality and missing data. *BayeScan* analyses using priors of 1,000 and 10,000 initially recovered seven putative loci under selection and after removing those loci (which were found throughout sample localities), a secondary run at both priors recovered no additional loci potentially under selection (Figure S1). Further, these loci were blasted against NCBI’s database but no significant matches were found, likely due to the short length of 2b‐RAD loci. Because *BayeScan* is known to suffer from type I errors (Beaumont & Balding, 2004; Narum & Hess, 2011), we opted to remove these seven loci likely under selection, as most population genetic analyses assume neutral loci, leaving 4,071 retained SNPs for all downstream analyses.

### 3.2 Genetic diversity, population structure, and gene flow

Observed and expected heterozygosity, along with inbreeding coefficients are reported in Table 1. All recovered observed heterozygosities were lower than the calculated expected heterozygosity, with the disjunct location Sainte‐Anne deviating by 17.56%, while all other sites averaged 14.53%. Sainte‐Anne also had the highest inbreeding coefficient at 0.196, with lower and upper limit confidence intervals of 0.183 and 0.208, respectively. Recovered genetic distance indices (Table 2) for all locations varied from 0.0081 to 0.0230 for Cavalli‐Sforza and Edwards Chord estimates and 0.0017 to 0.0396 for Weir and Cockerham's pairwise *F*_st_. Arcachon, which is centrally located in the middle of *D. biscayensis*’ geographic range in the Bay of Biscay (Figure 1), had some of the highest genetic differentiation values, 0.0230 and 0.0396, respectively; no other site had genetic diversities above 0.015 and 0.0133 for the respective pairwise comparisons. Additionally, Arcachon samples had the highest overall Cavalli‐Sforza and Edwards Chord distance recovered when compared with Sainte‐Anne, the most geographically isolated site in this study, 0.0230. Further, Archachon also had the highest Weir and Cockerham's pairwise *F*_st_ when compared to Tharon Plage, a value of 0.0396, with a lower limit confidence interval of 0.0369 and an upper limit confidence interval of 0.0424. Results of the AMOVA (Table 3) recovered the largest variance from individual samples within a locality as expected for panmictic populations, while differences among localities represented the least amount of variance, though both were highly significant. These tests were performed on the entire data set and also with Sainte‐Anne removed, as Sainte‐Anne is hypothesized to be a subset from the Bay of Biscay, but results only differed slightly between the two analyses. In both cases, differences among localities explained the least amount of the variance, 1.3% in both instances, consistent with little differentiation across the range.

**TABLE 2:** (A) Genetic distances calculated in *hierfstat* among the seven sampling localities of *Diopatra biscayensis*. (B) Weir and Cockerham's pairwise *F*_st_ distances bootstrapped over 1,000 replicates calculated in *hierfstat* among the seven sampling localities of *Diopatra biscayensis*

	Arcachon	Gatseau	Le Bile	Sainte‐Anne	Santander	San Vicente	Tharon Plage
(A)							
Arcachon	–	0.0208	0.0247	0.0292	0.0169	0.0176	0.0396
Gatseau	0.0150	–	0.0099	0.0062	0.0017	0.0049	0.0066
Le Bile	0.0184	0.0126	–	0.0132	0.0093	0.0116	0.0127
Sainte‐Anne	0.0230	0.0120	0.0145	–	0.0071	0.0092	0.0041
Santander	0.0151	0.0098	0.0134	0.0133	–	0.0060	0.0075
San Vicente	0.0126	0.0081	0.0119	0.0122	0.0097	–	0.0133
Tharon Plage	0.0216	0.0100	0.0129	0.0103	0.0114	0.0111	–
(B)							
Arcachon	–	0.0226	0.0271	0.0321	0.0186	0.0194	0.0424
Gatseau	0.0188	–	0.0116	0.0077	0.0030	0.0062	0.0080
Le Bile	0.0223	0.0084	–	0.01522	0.01070	0.01313	0.01448
Sainte‐Anne	0.0264	0.0048	0.0113	–	0.0087	0.0106	0.0052
Santander	0.0150	0.0004	0.0078	0.0054	–	0.0073	0.0091
San Vicente	0.0156	0.0037	0.0103	0.0077	0.0046	–	0.0148
Tharon Plage	0.0369	0.0053	0.0110	0.0029	0.0059	0.0117	–

Note

Distances below the diagonal were calculated under the default Cavalli‐Sforza and Edwards Chord distance, and distances above the diagonal were calculated using Weir and Cockerham's pairwise *F*_st_.

**TABLE 3:** AMOVA results for all sampling localities and with Sainte‐Anne removed

	All localities			All localities except Sainte‐Anne		
	Percent of variation	Obs	Std Obs	Percent of variation	Obs	Std Obs
Variation among inds relative to all samples	94.3	318.7	−5.2	94.0	359.8	−5.2
Variation among inds within a locality	4.4	14.8	4.1	4.7	17.9	4.0
Variation among localities	1.3	4.5	21.0	1.3	5.1	19.7

“Obs,” observed variance; “Std Obs,” standardized observation from Monte Carlo simulation; “inds,” individuals. All *p*‐values were significant at 0.001.

Both DAPC and *Admixture* analyses, which try to estimate the number of populations (*K)*, found that over multiple simulations *K* = 1 had the highest likelihood, with *K* = 2 having the second highest support. In conjunction with the analyses of genetic distances, DAPC and *Admixture* analyses were influenced by the genetically distinct Arcachon locality, with sharp drop‐offs in support for *K* = 2 when this location was removed from the data set. We conservatively estimate, henceforth, that *D. biscayensis*, given its geographic range from the Bay of Biscay to the Normanno‐Breton Gulf, is a singular population.

**FIGURE 2:**
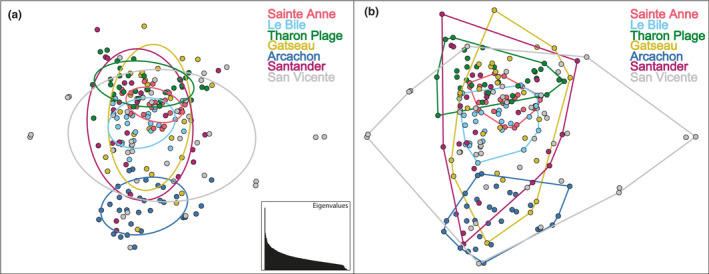
Genetic variation of SNP data for *Diopatra biscayensis*. (a) Principal component analyses of variation colored by sampling locality. (b) Convex hull analysis colored by locality with localities listed from north (Sainte‐Anne) to south (San Vicente). The furthest south localities of San Vicente and Santander retained the largest window space indicating higher genetic diversity at these sites. Sainte‐Anne is located at the center of the analyses, indicating that it is a subset of the biodiversity recovered in the Bay of Biscay

Principal component analyses (Figure 2a) recovered overlapping clusters from all sampling locations, further supporting that *D. biscayensis* is a singular population over this geographic range. Figure 2a presents PCA1 and PCA2, but other combinations of PCA1, PCA2, and PCA3 were tested and still recovered a singular population over the tested geographic range. Consistent with other analyses, PCA placed Arcachon as the most genetically distinct cluster, indicating a more limited connectivity with the surrounding areas. Sainte‐Anne, the most geographically isolated locality, consistently grouped in the center of all PCAs. Convex hull analyses (Figure 2b) showed Sainte‐Anne to occupy the smallest areal extent of PCA space (155), suggesting a reduced genetic diversity compared with other sites, followed by Le Bile (304), Tharon Plage (398), Arcachon (443), Gatseau (1,121), Santander (1,276), and finally San Vicente (2,640), suggesting that it had the most limited genetic diversity.

**FIGURE 3:**
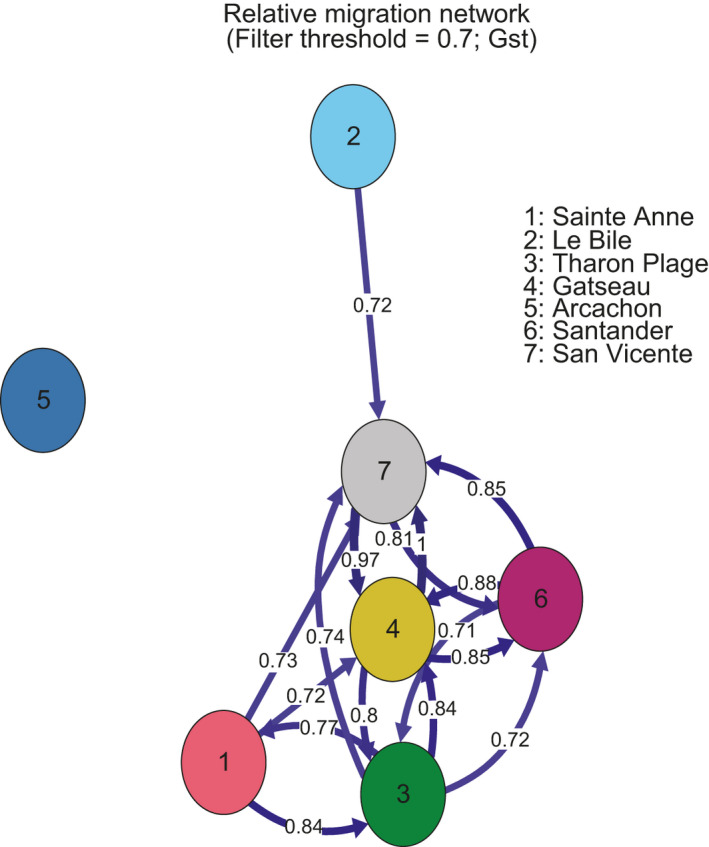
Relative migration network calculated between sampling localities using the statistic Gst and visualized with the software package *divMigrate*. Colors of sampling localities are consistent with Figures 1 and 2 for ease of comparison. Inferences made about directionality are limited due to human‐mediated dispersal

Estimates of migration rates and directionality calculations with Gst done in *divMigrate* further support these findings (Figure 3). With a moderate Gst cutoff of 0.7, we recovered no meaningful connections of Arcachon to its surrounding localities. Migration analyses were subsequently run without Arcachon to mitigate any impact on the inference between the remaining localities, but results did not differ. According to *divMigrate*, Le Bile, the furthest north locality in the Bay of Biscay, only showed connection with San Vicente, and Sainte‐Anne was most strongly connected with Tharon Plage. However, given that DAPC analysis, *Admixture* analysis, and PCA all show limited genetic structure between localities, we are reserved about the biological meaning of the *divMigrate* results. Calculating migration between genetically similar localities, with potential human‐mediated transport, is problematic.

**FIGURE 4:**
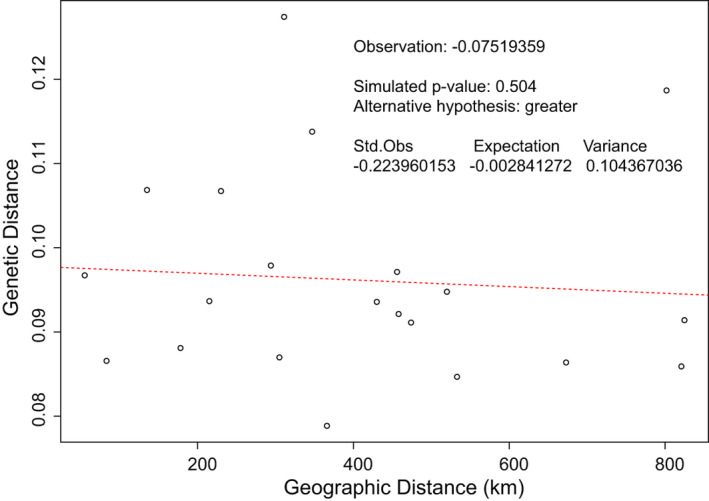
Mantel test of isolation by distance based on Cavalli‐Sforza and Edwards Chord distances between all seven sampling localities of *Diopatra biscayensis*. Tests were found to not be significant, suggesting that geographic distance over the sampling range is not a good predictor of genetic differentiation

All tests of IBD were found to not be significant, with most recovering a negative correlation. Given that Arcachon was a geographically central but genetically distinct locality, tests of IBD were performed with and without these individuals. The recovered IBD value was still negative at −0.075 and found to not be significant, with a p‐value of 0.504 (Figure 4).

## 4 DISCUSSION

Population structure analyses based on 4,078 SNP loci suggest the current distribution of *D. biscayensis* is consistent with the hypothesis of northward population expansion by human‐assisted dispersal (Wethey et al., 2016; Woodin et al., 2014). The genetic signature of the Sainte‐Anne population was a subset of the diversity found in the Bay of Biscay, supporting the notion that *D. biscayensis* was introduced to the Normanno‐Breton Gulf. Given the separation around Brittany, if the Sainte‐Anne's population had been a result of range contraction, then an isolation‐by‐distance (IBD) genetic signature would have been expected. An anthropogenic‐based introduction to the Bay of Mont‐Saint‐Michel is further supported by the fact that the lecithotrophic larvae of *D. biscayensis* have limited dispersal capability, <<50 km (Arias & Paxton, 2015; Woodin et al., 2014). The time needed to migrate across 450 km of unsuitable habitats and tidal fronts to naturally colonize the Normanno‐Breton Gulf is many times greater than the planktonic larval duration of this worm (Wethey et al., 2016). Sainte‐Anne also had the most limited diversity and represents a subset of the diversity recovered from the rest of the geographic range of *D. biscayensis* (PCA convex hull analyses), indicative of an introduction rather than an historic range contraction (Figure 2). An introduction of few founders followed by reproduction among those individuals would yield this result, similar to that seen in bays with limited water exchange (i.e., long water time residence), and thus facilitated autorecruitment (Ayata et al., 2009; Plus et al., 2009).

The two Spanish collection sites (Santander and San Vicente de la Barquera) retained the largest window space in the convex hull analysis (Figure 2b), indicating the highest genetic diversity among all studied localities. Interestingly, these localities correspond to the first records of *D. biscayensis* in Europe, dating back to the early 1900s (Arias & Paxton, 2015). Both are also locations with natural oyster banks that supported a considerable industry at that time (Torres & Corral, 2001). Given the long history of translocation of shellfish among regions in Europe (e.g., Goulletquer et al., 2002; Héral, 1989; Muehlbauer et al., 2014), and given the existence of an oyster fishery in San Vicente de la Barquera at least since the 1790s (Graells, 1870), an artisanal fishery in Santander in the 1860s (Graells, 1870), and a commercial fishery there in the 1890s (Dean, 1891), the development of commercial oyster culture in the Basque Country in the 1860s (Graells, 1870), and the use of oyster culturing methods from Arcachon in Spain (Balaguer y Primo, 1878), *D. biscayensis* plausibly dispersed from northern Spain with oyster transfers.

Migration analyses (Figure 3) suggested the geographically isolated Sainte‐Anne locality is more strongly connected to the Tharon Plage locality than to other sampling sites, but note that all connection values are >0.7 with the exception of Arcachon. As this relationship is unlikely through natural stepping stone dispersal across 450 km of inhospitably cold waters for *D. biscayensis’* reproduction (Wethey et al., 2016; Woodin et al., 2014), we considered possible anthropogenic vectors for this connectivity. Biofouling on ships is a common dispersal vector for many marine species (Floerl & Coutts, 2009), but the aquaculture industry remains a primary candidate for nontarget species dispersal through hitchhiking. Examples of polychaetes that overcame biogeographic barriers by human‐mediated transport in mollusk aquaculture are common (Naylor et al., 2001; Simon et al., 2009; Williams et al., 2016). Mussel seed transport to Northern Brittany and the Bay of Mont‐Saint‐Michel originated from the Bay of Biscay in 1954 and 1965, respectively, and continues to this day (Goulletquer & Le Moine, 2002). Important potential source populations are in the region between the Loire and Gironde rivers on the central French Biscay coast (Héral, 1989; Muehlbauer et al., 2014), which include very large populations of *D. biscayensis*. Ropes are put out into the intertidal for collection of seed and then transported by truck to grow‐out locations in the Bay of Mont‐Saint‐Michel and further north within the Bay of Biscay. Transport of oyster seed and adults follows similar pathways (Buestel et al., 2009; Goulletquer & Heral, 1997; Goulletquer, 1998; Goulletquer & Le Moine, 2002). Aquaculture materials are the likely transport mechanism given the finding of juvenile *Diopatra* on live mussel and oyster shells, ropes, etc. The long history of mussel and oyster aquaculture in France associated with these aquaculture practices makes this the likely vector for how *D. biscayensis* colonized the northern region of the species geographic range as suggested by Woodin et al. (2014). In the same way, another large‐sized errant polychaete *Marphysa victori*, originally described from Arcachon Bay in 2017 (Lavesque et al., 2017), is now considered to be an alien species in France (Lavesque et al., 2020). Based on morphological and molecular evidence, these authors have proposed an Asiatic origin of the species, considering that it was introduced from China or Japan into Arcachon Bay in the 1970s with the non‐native oyster *Magallana* (*Crassostrea*) *gigas*. Consistent with this notion, there is other abundant evidence for the association between aquaculture and the introduction of exotic species into Europe and secondary introductions among areas with aquaculture (Goulletquer & Le Moine, 2002; Mineur et al., 2007).

Although our interests originated with the connectivity of Sainte‐Anne locality to the rest of the Bay of Biscay, the genetic distinctness of Arcachon necessitates further investigation. This bay, while centrally located, shows signs of genetic differentiation from the rest of the geographic range of *D. biscayensis* and migration analyses did not uncover even moderate connectivity to the surrounding localities. The recovered substructure of Arcachon within the singular population is unusual given its central placement in the Bay of Biscay. Arcachon is a semi‐closed bay with aquaculture efforts focused on oyster farming and is a popular tourist location, increasing the potential for anthropogenic transport by the live bait industry. One possible explanation for the limited connectivity with surrounding localities is the unique hydrodynamics of Arcachon Bay. Specifically, river flows and wind‐driven currents have only minor impacts on the hydrology, instead tidal flows account for the majority of water mass movement, which are somewhat restricted by a sill near the mouth at ~ 20 m depth; most of the water masses re‐enter the bay after moving seaward on ebb tides (Plus et al., 2009). Plus et al. (2009) found that under ideal conditions with strong northerly and westerly winds, the flushing time for the bay ranged from 13.3 to 15.9 days; given that *D. biscayensis* larvae are in the water column for 4–5 days, this likely limits the species’ capabilities to disperse in or out of the bay. Strong currents have been shown to influence the recovered phylogeography with multiple other marine species (Collins et al., 2018; Galaska et al., 2017a; Xuereb et al., 2018). Some larval distribution data support the idea of domination by tidal advection in Arcachon Bay (Marcano & Cazaux, 1994; Mathivat‐Lallier & Cazaux, 1990). If currents are driving the genetic structure recovered at Arcachon, other species in the bay that have limited dispersal capabilities would also share similar phylogeographic patterns.

The single population of *D. biscayensis* recovered from the Bay of Biscay up to the Normanno‐Breton Gulf serves as another example of how aquaculture can affect natural populations and influence ecosystems. Introduced species often can have major impacts on the local ecosystem, causing competition for local resources, interrupting food webs, and leading to an economic impact (Leung et al., 2002). Even estimating the number of introductions can become problematic once the species has become established (Resh et al., 2018). *Diopatra biscayensis* is an ecosystem engineer that influences the overall habitat which it occupies and can have a major impact on the biodiversity within the region (Berke et al., 2010). Although habitat modification by *Diopatra* species can increase overall biodiversity for a locality, it also can result in displacement of endemic fauna via competition (Berke et al., 2010). Interestingly, the biology of *D. biscayensis* is strongly influenced by temperature (Berke et al., 2010; Wethey et al., 2011; Wethey et al., 2016) and the presumably introduced population in Sainte‐Anne is likely to expand given the continued progression of climate change.

## AUTHOR CONTRIBUTION

**Matthew P. Galaska:** Formal analysis‐Equal, Methodology‐Equal, Writing‐original draft‐Equal, Writing‐review & editing‐Equal; **David S**. **Wethey:** Conceptualization‐Equal, Funding acquisition‐Equal, Resources‐Equal, Writing‐review & editing‐Equal; **Andrés Arias:** Resources‐Equal, Writing‐review & editing‐Equal; **Stanislas** **F. Dubois:** Resources‐Equal, Writing‐review & editing‐Equal; **Kenneth M**. **Halanych:** Funding acquisition‐Equal, Methodology‐Equal, Writing‐review & editing‐Equal; **Sarah A**. **Woodin:** Conceptualization‐Equal, Funding acquisition‐Equal, Resources‐Equal, Writing‐review & editing‐Equal.

## CONFLICT OF INTEREST

None declared.

## Supporting information

Supplementary Material

## Data Availability

Specimen vouchers have been deposited at the Auburn University Museum of Natural History (Accession Numbers AUMNH 45517–45683). Raw sequences have been submitted to NCBI’s SRA database as BioProject PRJNA706051, Accession Nos. SAMN18115220‐SAMN18115428. The final SNP data set has been deposited at Dryad under project code https://doi.org/10.5061/dryad.4j0zpc8b4.
