# Supplementary material for: The impact of aquaculture on the genetics and distribution of the onuphid annelid Diopatra biscayensis
Source: Ecol Evol. 2021 May 2;11(11):6184–94. doi: 10.1002/ece3.7447 (PMC8207402; doi:10.1002/ece3.7447)
Supplement: Supplementary file 1 — Supplementary Material [file ECE3-11-6184-s001.docx]

SUPPORTING INFORMATION


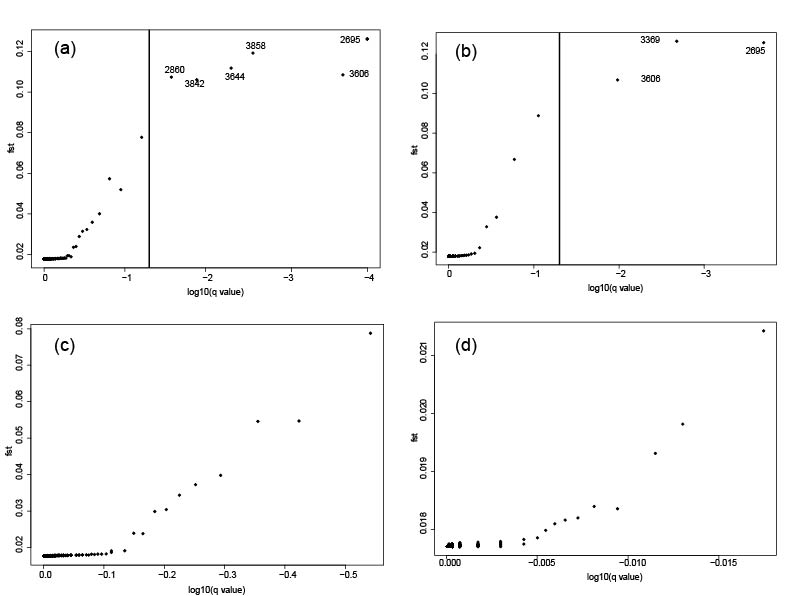


Supplemental Figure 1. BayeScan simulations to identify loci possibly under selection. Simulations had 50,000 burn-in steps with an additional 100,000 intervals with priors set at A) 1,000 with all loci, B) 10,000 with all loci, C) 1,000 with loci under possible selection removed and D) 10,000 with loci under possible selection removed.

|  | *K*=1 | *K*=2 | *K*=3 | *K*=4 | *K*=5 |
| --- | --- | --- | --- | --- | --- |
| DAPC (BIC) | 1314.9 | 1315.9 | 1318.7 | 1322.1 | 1325.1 |
| Admixture (CV) | 0.5416 | 0.5445 | 0.5444 | 0.5479 | 0.5489 |

Supplemental Table 1. Selection of *K* statistics from DAPC (Bayesian Information Criterion values) and Admixture (Cross-validation values). For both analyses, a lower number indicates higher statistical support for the number of populations. The table presents the final simulations which are representative of the majority of iterations where *K*=1. Due to the high number of simulations repeated, a small fraction found *K*=2 with the highest probability. Discussed in the main manuscript, Arcachon’s genetic differentiation appears to drive this variability.
